# Emotional Eating Phenotype is Associated with Central Dopamine D2 Receptor Binding Independent of Body Mass Index

**DOI:** 10.1038/srep11283

**Published:** 2015-06-12

**Authors:** Sarah A. Eisenstein, Allison N. Bischoff, Danuta M. Gredysa, Jo Ann V. Antenor-Dorsey, Jonathan M. Koller, Amal Al-Lozi, Marta Y. Pepino, Samuel Klein, Joel S. Perlmutter, Stephen M. Moerlein, Kevin J. Black, Tamara Hershey

**Affiliations:** 1 Departments of Psychiatry, Washington University School of Medicine, St. Louis, MO 63110, USA; 2 Departments of Radiology, Washington University School of Medicine, St. Louis, MO 63110, USA; 3 Departments of Internal Medicine, Washington University School of Medicine, St. Louis, MO 63110, USA; 4 Departments of Neurology, Washington University School of Medicine, St. Louis, MO 63110, USA; 5 Departments of Anatomy & Neurobiology, Washington University School of Medicine, St. Louis, MO 63110, USA; 6 Departments of Physical Therapy, Washington University School of Medicine, St. Louis, MO 63110, USA; 7 Departments of Occupational Therapy, Washington University School of Medicine, St. Louis, MO 63110, USA; 8 Departments of Biochemistry and Molecular Biophysics, Washington University School of Medicine, St. Louis, MO 63110, USA

## Abstract

PET studies have provided mixed evidence regarding central D2/D3 dopamine receptor binding and its relationship with obesity as measured by body mass index (BMI). Other aspects of obesity may be more tightly coupled to the dopaminergic system. We characterized obesity-associated behaviors and determined if these related to central D2 receptor (D2R) specific binding independent of BMI. Twenty-two obese and 17 normal-weight participants completed eating- and reward-related questionnaires and underwent PET scans using the D2R-selective and nondisplaceable radioligand (*N*-[^11^C]methyl)benperidol. Questionnaires were grouped by domain (eating related to emotion, eating related to reward, non-eating behavior motivated by reward or sensitivity to punishment). Normalized, summed scores for each domain were compared between obese and normal-weight groups and correlated with striatal and midbrain D2R binding. Compared to normal-weight individuals, the obese group self-reported higher rates of eating related to both emotion and reward (*p* < 0.001), greater sensitivity to punishment (*p* = 0.06), and lower non-food reward behavior (*p* < 0.01). Across normal-weight and obese participants, self-reported emotional eating and non-food reward behavior positively correlated with striatal (*p* < 0.05) and midbrain (*p* < 0.05) D2R binding, respectively. In conclusion, an emotional eating phenotype may reflect altered central D2R function better than other commonly used obesity-related measures such as BMI.

Reward-related behavioral and neurocircuitry dysfunction may contribute to obesity^1^ and provide therapeutic targets for prevention and treatment of the disease. However, the role of striatal dopamine (DA) signaling in human obesity remains unclear due to mixed results of PET/SPECT studies that assess the relationship between body mass index (BMI) and D2/D3 DA receptor (D2/D3R) availability. Some studies have found that striatal D2/D3R availability is lower in obesity and negatively correlates with BMI^2^,^3^,^4^ while others find no difference^5^,^6^,^7^ or higher D2/D3R availability in obese versus normal weight individuals^8^ or with increasing BMI^9^. Using a highly specific and non-displaceable ligand, we found no significant associations of D2 receptor subtype (D2R) binding with obesity or BMI^10^.

Differences in human obesity DA PET study findings may be due to several factors. For instance, the study samples used have had different degrees of obesity, ranging from overweight (BMI 25.0-29.9 kg/m^2^)^3^,^6^,^9^ and mild Class I (BMI 30.0-34.9 kg/m^2^)^3^ obesity to more severe Class III (BMI ≥ 40.0 kg/m^2^)^2^,^4^,^5^,^8^,^9^,^10^ obesity. Obesity phenotype and DA signaling abnormalities may differ across classes of obesity^1^,^6^. To further complicate interpretation, most of these studies employed radioligands with important limitations. Specifically, [^11^C]raclopride and [^18^F]fallypride do not distinguish between D2R and D3R^11^, which are localized differently across the brain and may be functionally distinct^12^. Furthermore, these radioligands are displaceable by DA, so D2/D3R availability measures are influenced by endogenous DA release as well as by D2/D3R binding *per se*^13^,^14^,^15^.

Although BMI is not consistently correlated with D2/D3R availability^16^, behavioral aspects of obesity may have a closer relationship to DA signaling. To address this issue and the limitations described above, we assessed obesity-associated characteristics that may relate to DA signaling, such as emotion- and reward-based eating and behavior motivated by non-food reward and sensitivity to punishment, in obese and normal weight participants. We investigated whether these characteristics correlated with striatal D2R using (*N*-[^11^C]methyl)benperidol ([^11^C]NMB), a PET radioligand DA D2 receptor antagonist that is highly selective for D2R over D3R^17^ and other G-protein receptors and is not displaced by endogenous DA release^18^. In addition, since novelty seeking behavior is associated with midbrain D2/D3R binding^19^, we explored the relationship between midbrain D2R binding and obesity-associated behavior.

## Methods

### Participants

Participants included 17 normal-weight and 22 obese individuals (see Table 1). One individual in the normal-weight group was slightly overweight (BMI = 25.9 kg/m^2^) but percent body fat and other weight parameters met normal-weight criteria. Selected data from 15 participants from each group were reported previously^10^. After an overnight fast (at least 8 hr), participants underwent comprehensive medical evaluation, routine blood tests, hemoglobin A1C, and an oral glucose tolerance test (OGTT). Individuals with self-reported history of diabetes, A1C ≥ 6.5%, or OGTT results that indicated impaired fasting glucose, impaired oral glucose tolerance, or diabetes were excluded. Individuals were also screened and excluded for IQ < 80^20^ (WASI), and conditions including parkinsonism, lifetime psychosis, mania, substance dependence, major depression, social phobia, eating disorders (including binge eating disorder) and panic disorder by neurological examination and psychiatric interview (Structured Clinical Interview for DSM-IV^21^). Current smoking and medications related to DA function were also exclusionary. No participant had smoked tobacco during the past 11 mos. or used medications related to DA function during the past month. All participants provided written informed consent. All procedures were performed in accordance with the Declaration of Helsinki and approved by Washington University’s Human Research Protection Office and Radioactive Drug Research Committee.

### Questionnaires

During the day of the OGTT, immediately and 1 hr after which a light snack and lunch were provided, respectively, participants completed questionnaires addressing DA-related constructs, or domains, of interest: 1) eating behavior related to emotion including avoidance of negative affect; 2) eating behavior related to reward including craving for palatable foods and inability to limit intake of sweet foods; 3) non-food reward behavior, including approach, sensitivity, motivation, and expectancy for non-food reward stimuli; and 4) punishment avoidance including inhibition, sensitivity, and expectancy. Self-report questionnaires or subscales of self-report questionnaires were included in these different domains (Table 2) based on their descriptions in original manuscripts introducing and validating the questionnaire. Scores for each questionnaire or subscale were converted into *z*-scores and summed together with other measures included in the domain to yield final domain scores for each individual.

The following questionnaires were included in the Eating Related to Emotion domain: The Emotional Eating Scale^22^ (EES) assesses urge to eat due to negative emotion. The Dutch Eating Behavior ‘Emotional’ subscale^23^ (DEBQ ES) consists of summed self-ratings of tendencies to eat in response to both ‘diffuse’ (e.g., bored) and ‘clearly labeled’ (e.g., anger) emotions. The ‘mood-altering effect’ subscale of the Sweet Taste Questionnaire^24^ (STQ MAE) assesses the degree to which eating sweet foods alters mood in a positive manner.

The following questionnaires were included in the Eating Related to Reward domain: The Binge Eating Scale^25^ (BES) assesses the degree to which one experiences binge eating, including behavior (e.g., eating in secret) and emotions that occur before and after a binge (e.g. lack of control). The ‘Impaired control over eating sweets’ subscale of the STQ^24^ (STQ IC) is a measure of one’s ability to refrain from eating sweets. We used the total score on the Food Craving Inventory^26^ (FCI) to measure general craving for sweet and high-carbohydrate or fatty foods.

The following questionnaires were included in the Non-food Reward domain: The Behavioral Activation System (BAS) portion of the BIS/BAS^27^ questionnaire consists of three subscales: drive, fun-seeking and reward responsiveness. It is meant to measure BAS sensitivity. Individuals with stronger BAS should be more sensitive and derive more pleasure upon exposure to reward cues^28^,^29^. The sensitivity to reward portion of the Sensitivity to Punishment and Sensitivity to Reward Questionnaire^30^ (SPSRQ) also assesses BAS functioning. The reward expectancy portion of the Generalized Reward and Punishment Expectancy Scales^31^ measures optimism and expectancy of positive life events. The ‘curiosity behavior’, or novelty-seeking, dimension of the Temperament and Character Inventory^32^ (TCI-R) reflects bias towards active novelty-seeking, impulsivity and approach towards reward cues. The ‘reward dependency’ dimension of the TCI reflects bias towards prosocial behavior and social approval. The ‘persistence’ dimension of the TCI reflects degree of perseverance despite fatigue and other obstacles.

The following questionnaires were included in the Punishment domain: The Behavioral Inhibition System (BIS) portion of the BIS/BAS^27^ questionnaire measures BIS sensitivity. People with stronger BIS sensitivity should be more sensitive to and experience greater anxiety in response to punishment cues^28^,^29^. The punishment portions of the GRAPES^31^ and SPSRQ^30^ assess punishment expectancy and sensitivity, respectively. The ‘damage avoidance’ section of the TCI-R^32^ assesses bias towards behavior aimed at avoiding harm.

### MRI and PET Acquisition

On a day separate from the day of the OGTT, participants underwent MRI and 2 hr PET scans, which took place between 0900 and 1700. Methods for [^11^C]NMB synthesis, MRI and PET scan acquisitions are described previously^10^. Each participant intravenously received 6.4 – 18.1 mCi containing <7.3 μg unlabeled NMB. [^11^C]NMB purity was ≥96% and specific activity ≥1066 Ci/mmol (39 TBq/mmol). Since [^11^C]NMB is not displaceable by endogenous DA^18^, participants were not asked to fast or otherwise modify their food intake on the night before or day of the scans.

### ROI-based Analyses

The methods for our ROI-based analyses are described in Eisenstein *et al.*^10^,^33^. The neuroimaging software FreeSurfer (http://surfer.nmr.mgh.harvard.edu) was used for segmentation of striatal regions^34^. To limit multiple comparisons, D2R specific binding (BP_ND_) for each ROI was averaged across left and right hemispheres. Putamen and caudate D2R BP_ND_s were averaged to obtain a composite dorsal striatal BP_ND_ and ventral striatal BP_ND_ included average nucleus accumbens D2R BP_ND._ The midbrain region was traced on each individual’s MPRAGE as previously described^33^.

### Voxel-based Analyses

We conducted voxel-based analyses to determine whether specific striatal or midbrain clusters of D2R binding related to BMI or Eating Related to Emotion, Eating Related to Reward, Non-food Reward, and Punishment behavioral domain scores. Images of D2R BP_ND_ across the brain were generated for each participant and smoothed with a 6 mm full width at half maximum kernel. These images were averaged across normal-weight and obese individuals and thresholded at BP_ND_ = 0 to use as an explicit mask for regions including striatum or subcortical regions only. Positive and negative associations between D2R binding and dependent variables were tested at the voxel level using SPM8 (http://www.fil.ion.ucl.ac.uk/spm).

### Primary Statistical Analyses

Much of the data was managed using REDCap electronic data capture tools hosted by the Biostatistics Division of Washington University School of Medicine^35^. Group demographic variables were compared with Pearson Chi Square, Mann Whitney *U*, or *t*-tests. Dorsal and ventral striatal BP_ND_ were compared with repeated measures ANCOVA covarying for age, ethnicity, and education. Midbrain D2R BP_ND_ and domain scores were compared between normal-weight and obese groups with ANCOVAs covarying for age, ethnicity and education. Significant findings for a behavioral domain were followed up with exploratory ANCOVAs of each questionnaire contributing to that domain. Separate hierarchical linear regression models with appropriate covariates (age, ethnicity, education level, and/or BMI) were used to analyze the ability of each variable of interest to predict striatal or midbrain D2R BP_ND_. These analyses also yielded partial correlations describing the unique variance contributed by each variable of interest to BP_ND._ For voxelwise analyses, correlations between D2R binding and BMI and behavioral domain scores were calculated as Pearson’s *r* and tested for significance with Student’s one-sample *t*-tests covaried for age, ethnicity, education, and, for behavioral domains, BMI, at each voxel. For SPM analyses, *p* ≤ 0.001, after multiple comparison correction, at voxelwise level was considered significant. For all other analyses, significance level was set at α ≤ 0.05.

## Results

### Participant Characteristics

Normal-weight and obese groups are described in Table 1. We did not have complete Non-food Reward and Punishment questionnaire datasets from one obese individual and another obese individual did not undergo a PET scan. Therefore, analyzed data sets including these variables consist of 21 obese and 17 normal-weight individuals. One normal-weight participant’s midbrain D2R BP_ND_ was too low to be quantified by our processing software and analyses including this variable included 20 or 21 obese and 16 normal-weight participants.

### BMI and Central D2R Specific Binding

As in our previous report on a subset of these individuals^10^, after covarying for age, ethinicity and education level, obese and normal-weight groups did not differ in striatal BP_ND_ (normal weight mean total striatal BP_ND_ = 10.30, S.D. = 1.17; obese mean total striatal BP_ND_ = 10.22, S.D. = 1.34; *F*_1,33_ = 1.98, *p* = 0.17). Across both groups, dorsal striatal D2R BP_ND_ was greater than ventral striatal BP_ND_ at a marginally significant level (dorsal mean BP_ND_ = 4.09, S.D. = 0.52; ventral mean BP_ND_ = 2.08, S.D. = 0.29; *F*_1,33_ = 3.87, *p* = 0.06) and there was no significant interaction between group and striatal region (*F*_1,33_ = 1.98, *p* = 0.17). Midbrain D2R BP_ND_ was not different between normal-weight and obese groups (normal-weight mean BP_ND_ = 0.27, S.D = 0.14; obese mean BP_ND_ = 0.27, S.D. = 0.09; *F*_1,32_ = 0.15, *p* = 0.70).

**Figure 1:**
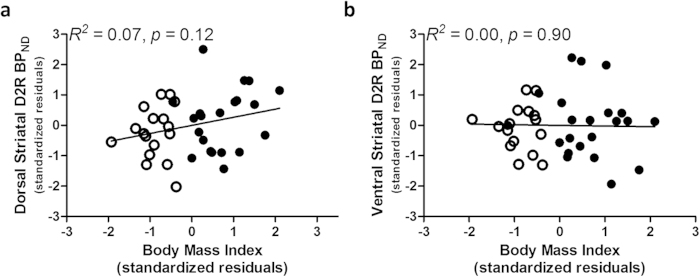
BMI and striatal D2R are not significantly correlated across normal-weight (clear circles) and obese (filled circles) groups. D2R specific binding was not correlated with BMI in the **a**, dorsal or **b**, ventral striatum. Data points are standardized residuals of partial correlations after covarying for age, ethnicity and education level. BP_ND_, dopamine D2 receptor binding potential.

Controlling for age, ethnicity and education, BMI did not predict striatal BP_ND_ across all participants (dorsal *R*^*2*^ change = 0.07. *F*_1,33_ = 2.61, *p* = 0.12; ventral *R*^*2*^ change = 0.00. *F*_1,33_ = 0.02, *p* = 0.90) (Fig. 1), or within either group (normal-weight: dorsal *R*^*2*^ change = 0.01; *F*_1,12_ = 0.19, *p* = 0.67, ventral *R*^*2*^ change = 0.00. *F*_1,12_ = 0.002, *p* = 0.97; obese: dorsal *R*^*2*^ change = 0.03; *F*_1,16_ = 0.62, *p* = 0.44, ventral *R*^*2*^ change = 0.04; *F*_1,16_ = 0.99, *p* = 0.33). Similarly, BMI did not predict midbrain D2R BP_ND_ across normal-weight and obese participants (*R*^*2*^ change = 0.00. *F*_1,32_ = 0.00_1,_ *p* = 0.98) or within either group (normal-weight: *R*^*2*^ change = 0.05; *F*_1,11_ = 0.55, *p* = 0.48; obese: *R*^*2*^ change = 0.12; *F*_1,16_ = 2.51, *p* = 0.13).

### Obesity-associated Behavior

Table 2 presents group mean (S.D.) summed *z*-scores for each domain and raw scores for each questionnaire.

**Figure 2:**
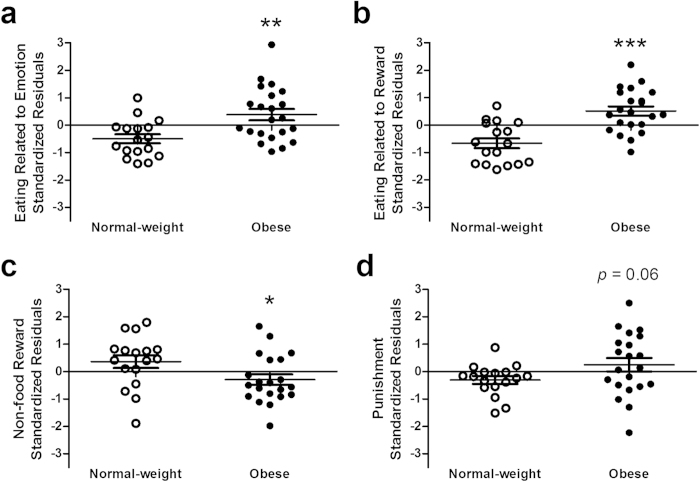
Behaviors thought to be tightly coupled to dopamine signaling differ between normal-weight and obese individuals. Obese individuals self-report higher rates of **a,** emotion- and **b,** reward-based eating behavior, **c,** lower rates of non-food reward behavior, and **d,** higher rates of avoidance of punishment relative to normal-weight individuals. Data points are standardized residuals of partial correlations after controlling for age, ethnicity, and education level. *, **, ***, *p* ≤ 0.05, 0.01, 0.001 relative to normal-weight. For avoidance of punishment, *p* = 0.06 relative to normal-weight.

The obese group had higher mean domain scores on Eating Related to Emotion (*F*_1,34_ = 11.62, *p* < 0.01; Fig. 2A) and Eating Related to Reward (*F*_1,34_ = 28.47, *p* < 0.001; Fig. 2B) and a lower mean domain score on Non-food Reward (*F*_1,33_ = 5.37, *p* = 0.03; Fig. 2C). Punishment domain scores were higher in obese relative to normal-weight at a marginally significant level (*F*_1,33_ = 3.69, *p* = 0.06; Fig. 2D).

Within the Eating Related to Emotion domain, scores on all three questionnaires were correlated with each other (0.63 ≤ *r*_39_* ≤ *0.80, *p *< 0.001) and the obese group scored significantly higher than the normal-weight group on EES (*F*_1,33_ = 6.42, *p* = 0.02) and DEBQ ES (*F*_1,33_* = *4.75, *p* = 0.04) and marginally significantly higher on STQ MAE (*F*_1,33_* = *3.48, *p* = 0.07). BMI was associated with the summed domain score across the entire sample (*r*_39_ = 0.46, *p* < 0.01) but not when examined just within obese (*r*_22_ = −0.24, *p* = 0.29) or normal-weight (*r*_17_ = 0.09, *p* = 0.74).

The *z*-scores on the three questionnaires included in the Eating Related to Reward domain were correlated with each other (*r*_39_ = 0.43, *p* ≤ 0.01). The obese group scored higher on the BES (*F*_1,34_ = 19.57, *p* < 0.001), STQ IC (*F*_1,34_ = 14.77, *p* = 0.001) and the FCI (*F*_1,34_ = 10.35, *p* = 0.003). BMI related to the summed domain score in the entire sample (*r*_39_ = 0.37, *p < *0.02) but not within obese (*r*_22_ = 0.07, *p* = 0.78) or no*r*mal-weight (*r*_17_ = −0.03, *p* = 0.91).

**Figure 3:**
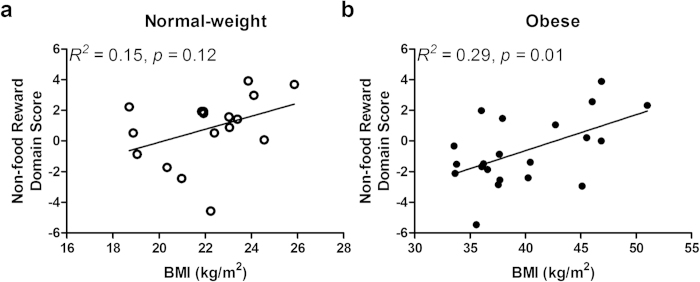
Although the obese group self-reported lower rates of non-food reward behavior relative to the normal-weight group, higher BMI was associated with higher rates of non-food reward behavior within obese individuals. **a**, BMI and non-food reward behavior, **a** composite measure of reward-related behavior including approach, expectancy and sensitivity towards reward stimuli other than food, was not significantly associated with BMI in the normal-weight group. **b**, Across obese individuals, there was a positive relationship between BMI and non-food reward behavior. BMI, body mass index.

Within the Non-food Reward domain, the individual questionnaires did not correlate (0.03 ≤ *r*_38_* ≤ *0.28, *p* ≥ 0.09). The obese group had a lower mean score than the normal-weight group on the behavioral approach subscale of the BIS/BAS (*F*_1,33_ = 6.47, *p* = 0.02). Groups did not differ significantly on any of the other Reward domain scales (SPSRQ: *F*_1,33_ = 0.21, *p* = 0.65; TCI-R: *F*_1,33_ = 0.44, *p* = 0.51) except at a marginally significant level on the GRAPES reward expectancy subscale (obese < normal-weight, *F*_1,33_ = 3.25, *p* = 0.08). BMI did not correlate significantly with the summed domain score in the entire sample (*r*_38_ = −0.11, *p = *0.51) or within normal-weight (*r*_17_ = 0.39, *p* = 0.12; Fig. 3A). However, BMI was correlated with the reward summed domain score within obese (*r*_21_ = 0.54, *p = *0.01; Fig. 3B).

Within the Punishment domain, scores on all questionnaires were correlated with each other (0.54 ≤ *r*_39_* ≤ *0.79, *p* ≤ 0.001). The obese group tended to score higher on the behavioral inhibition portion of the BIS/BAS (*F*_1,33_ = 3.11, *p* = 0.09) and the damage avoidance subscale of the TCI-R (*F*_1,33_ = 3.17, *p* = 0.08) than the normal-weight group; these differences were marginally significant. Obese and normal-weight groups did not differ on the punishment expectancy subscale of the GRAPES (*F*_1,33_ = 1.10, *p* = 0.30) or sensitivity to punishment subscale of SPRSQ (*F*_1,33_ = 2.30, *p* = 0.14). BMI did not correlate significantly with the summed domain score in the entire sample (*r*_38_ = 0.15, *p = *0.37) or within normal-weight (*r*_17_ = 0.21, *p* = 0.43) or obese (*r*_21_ = −0.35, *p = *0.12) groups.

### Obesity-associated Behavior and Central D2R BP_ND_

**Figure 4:**
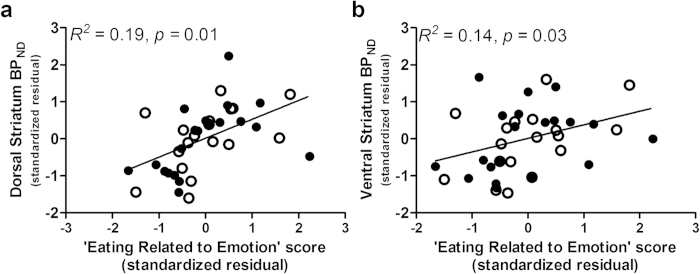
Self-reported emotional eating correlates with striatal D2R binding independent of BMI across normal-weight (clear circles) and obese (filled circles) individuals. Higher rates of emotion-based eating, **a** composite measure of self-reported tendency to eat to avoid negative emotion, was related to higher **a**, dorsal and **b**, ventral striatal D2R across normal-weight and obese groups. Data points are standardized residuals of partial correlations after controlling for age, ethnicity, education level, and BMI. BP_ND_, dopamine D2 receptor binding potential.

After covarying age, ethnicity, education level, and BMI, the Eating Related to Emotion domain score related to *dorsal* striatal BP_ND_ (*R*^*2*^ change = 0.13. *F*_1,32_ = 7.51, *p* = 0.01; partial *r* = 0.44; Fig. 4A) but Eating Related to Reward (*R*^*2*^ change = 0.02. *F*_1,32_ = 1.15, *p* = 0.29), Non-food Reward (*R*^*2*^ change = 0.01. *F*_1,31_ = 0.31, *p* = 0.58) and Punishment (*R*^*2*^ change = 0.00. *F*_1,31_ = 0.06, *p* = 0.81) domain scores did not. Within the Eating Related to Emotion domain, EES (*R*^*2*^ change = 0.08. *F*_1,32_ = 5.48, *p* = 0.03, *p*artial *r* = 0.38), DEBQ ES (*R*^*2*^ change = 0.12. *F*_1,32_ = 6.88, *p* = 0.01, *p*artial *r* = 0.42) and STQ MAE (*R*^*2*^ change = 0.10. *F*_1,32_ = 4.48, *p* = 0.04, *p*artial *r* = 0.35) scores were associated with dorsal striatal BP_ND_ .

After covarying age, ethnicity, education level, and BMI, Eating Related to Emotion domain scores (*R*^*2*^ change = 0.11. *F*_1,32_ = 5.18, *p* = 0.03) related to *ventral* striatal BP_ND_ (Fig. 4B) but Eating Related to Reward (*R*^*2*^ change = 0.05. *F*_1,32_ = 2.33, *p* = 0.14), Non-food Reward (*R*^*2*^ change = 0.00. *F*_1,31_ = 0.19, *p* = 0.67) and Punishment (*R*^*2*^ change = 0.02. *F*_1,31_ = 0.72, *p* = 0.40) domain scores did not. Within the Eating Related to Emotion domain, DEBQ ES (*R*^*2*^ change = 0.10. *F*_1,32_ = 4.71, *p* = 0.04, partial *r* = 0.36) scores significantly correlated with ventral striatal BP_ND_. STQ MAE (*R*^*2*^ change = 0.08. *F*_1,32_ = 3.93, *p* = 0.06; partial *r* = 0.33) and EES (*R*^*2*^ change = 0.07. *F*_1,32_ = 3.17, *p* = 0.09; partial *r* = 0.33) scores correlated with ventral striatal BP_ND_ at a marginally significant level.

**Figure 5:**
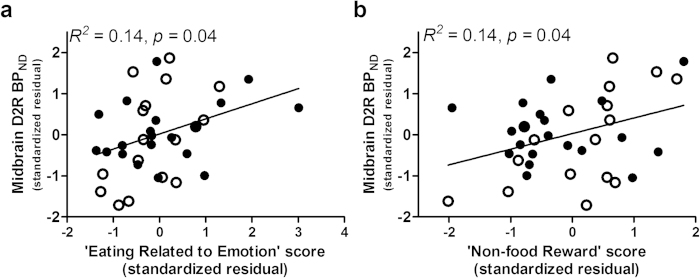
Midbrain D2R binding correlates with self-reported reward-related and eating behavior independent of BMI across normal-weight (clear circles) and obese (filled circles) individuals. **a**, Higher rates of non-food reward behavior, **b** composite measure of self-reported tendency to approach, anticipate, and/or be sensitive to rewarding stimuli other than food, was related to higher midbrain D2R across normal-weight and obese groups. **a**, Similar to striatal D2R binding, midbrain D2R binding positively related to self-reported emotional eating. Data points are standardized residuals of partial correlations after controlling for age, ethnicity, education level, and BMI. BP_ND_, dopamine D2 receptor binding potential.

After covarying age, ethnicity, education level, and BMI, midbrain D2R BP_ND_ was related to Eating Related to Emotion domain scores (*R*^*2*^ change = 0.10. *F*_1,31_ = 4.88, *p* = 0.04; partial *r* = 0.37, Fig. 5A). Within this domain, higher midbrain D2R BP_ND_ significantly related to higher EES (*R*^*2*^ change = 0.14. *F*_1,31_ = 6.48, *p* = 0.02; partial *r* = 0.42) and DEBQ ES (*R*^*2*^ change = 0.09. *F*_1,31_ = 4.71, *p* = 0.04; partial *r* = 0.36) scores but was not related to STQ MAE (*R*^*2*^ change = 0.03. *F*_1,31_ = 1.23, *p* = 0.28) scores. Midbrain D2R BP_ND_ was also related to Non-food Reward domain scores (*R*^*2*^ change = 0.13. *F*_1,30_ = 4.82, *p* = 0.04; partial *r* = 0.37, Fig. 5B). Within the Non-food Reward domain, higher midbrain D2R BP_ND_ related to higher scores on the BAS (*R*^*2*^ change = 0.10. *F*_1,30_ = 3.83, *p* = 0.06; partial *r* = 0.34) and reward sensitivity subscale of the SPSRQ (*R*^*2*^ change = 0.09. *F*_1,30_ = 3.73, *p* = 0.06; partial *r* = 0.33) at marginally significant levels but were not associated with scores on the reward expectancy subscale of the GRAPES (*R*^*2*^ change = 0.01. *F*_1,30_ = 0.30, *p = *0.59 ) or reward-related TCI-R scales (*R*^*2*^ change = 0.02. *F*_1,30_ = 0.78, *p* = 0.38). Midbrain D2R BP_ND_ was not associated with Eating Related to Reward (*R*^*2*^ change = 0.00. *F*_1,31_ = 0.01, *p* = 0.93) or Punishment (*R*^*2*^ change = 0.00. *F*_1,3_ = 0.05, *p* = 0.83) domain scores.

### Voxel-based Analysis

While positive BP_ND_-behavioral relationships appeared to be present in striatum and midbrain at less stringent criterion for statistical significance, there were no significant relationships observed between D2R binding and BMI or any of the behavioral domain scores at the voxel-wise level (*p* > 0.001 for all tests).

## Discussion

Our current findings contribute to the obesity and neuroimaging literature in several important ways. First, we characterize four different types of putatively DA-related behavior in rigorously screened, moderately obese and normal-weight participants using well-validated and reliable questionnaires. To our knowledge, no other study has investigated these behaviors simultaneously in obese and normal-weight individuals to the same extent. Second, our D2R binding measurements are not confounded by D3R binding and competition with endogenous DA because we used the relatively novel radioligand [^11^C]NMB, which is unique due to its high affinity and selectivity for D2R that appears impervious to endogenous DA. These radioligand properties permit us to quantify D2R specific binding levels rather than D2/D3R availability and avoid the influence of endogenous DA levels. Finally, we detected relationships between D2R binding and behavioral phenotypes, as measured by several validated and reliable self-report questionnaires. These relationships were specific to two of four behavioral domains we investigated and were independent of BMI. Moreover, BMI itself did not correlate with D2R specific binding. These data underscore the complex interaction among eating- and reward-related behavior, BMI, and measures of a key central reward system (striatal and midbrain D2R specific binding). Our findings that eating- and reward-related behavior linearly relate to striatal and midbrain D2R, respectively, support the notion that regulation of food intake and reward-driven behavior involve a central reward, motor, and habit formation system, even though D2R specific binding was not associated with BMI.

With our ROI-based analyses, we demonstrate that obesity-associated behavior, specifically self-reported higher rates of eating to avoid negative emotion, correlates with higher striatal D2R binding *in vivo* across obese and normal-weight participants, independent of BMI. This finding is consistent with the recent report that striatal D2/D3R availability is positively associated with a dimension of the Three-factor Eating Questionnaire, ‘opportunistic eating’^9^, which reflects habitual, emotional, and situational susceptibility to disinhibited eating^36^. Our finding is consistent with theirs, but extends the results by using several validated questionnaires related to emotional eating and a D2-selective radioligand. Our results are also in line with those of a study that showed multi-locus genetic profile scores reflecting enhanced DA function (including the *ANKK* single nucleotide polymorphism associated with D2R levels) relate to more emotional and binge eating^37^. Our findings differ from Volkow *et al.*^38^ in which greater emotionality was associated with *lower* dorsal striatal D2/D3 receptor availability. However, only nonobese participants were studied by Volkow *et al.*^38^ and screening criteria and the properties of the PET radioligand used were different than those in our study. Although not statistically significant, higher dorsal striatal D2R binding in our sample tended to relate to *higher* BMI across normal-weight and moderately obese individuals, similar to Dunn *et al.*^8^. Perhaps, as others propose^1^,^6^,^7^, striatal DA system overactivity induced by repeated emotional overeating in less severe forms of overweight or obesity eventually downregulates striatal D2/D3R, presenting as lower receptor availability in extremely obese individuals as in Wang *et al.*^4^ and de Weijer *et al*^2^. Alternatively, obese individuals with relatively higher striatal D2R binding may be protected from developing more severe forms of obesity. Unfortunately, scanner weight-limits and size of the bore precluded inclusion of severely or morbidly obese individuals in the current study. Future investigations should employ longitudinal and/or cross-sectional studies to determine whether striatal D2R and obesity-associated behaviors change in accordance with large changes in BMI (i.e. from moderate to severe obesity).

Our ROI-based analyses also showed that *midbrain* D2R binding related to self-reported emotional eating and non-food reward-related behavior in a positive manner across normal-weight and obese groups. This is not surprising given the midbrain’s roles in motivation, habit formation^39^, and activity geared towards obtaining reward^40^. Our results are in apparent contrast with those of Savage *et al.*^19^, in which a *negative* relationship between novelty-seeking and substantia nigra D2/D3R availability, as measured by [^18^F]fallypride, was observed in normal-weight but not obese individuals. However novelty-seeking was not specifically addressed in our study – it comprised one subscale of the TCI-R questionnaire. In addition, unlike the D2R-selective [^11^C]NMB, [^18^F]fallypride binds to both D2R and D3R and is sensitive to competition with endogenous DA^41^. Our results are in agreement with those of a previous study in which higher trait motivation related to higher midbrain and ventral striatal D2/D3R availability as measured by [^11^C]raclopride^42^. In our study, the relationship between midbrain D2R and non-food reward-related behavior appears to be driven by scores on the BAS^27^ and the SPSRQ^30^, which are meant to reflect responsivity to and drive for reward and reward sensitivity, respectively. In contrast to striatal D2R, midbrain D2R are thought to be almost exclusively located presynaptically and, when activated by DA transmission arising locally and from afferent projections, function as inhibitory receptors on cell bodies and dendrites of dopaminergic neurons, resulting in decreased DA release in midbrain and striatum^43^,^44^,^45^,^46^. Therefore, the midbrain may modulate DA transmission in mesostriatal reward circuitry through this negative feedback loop^45^. Since we observed positive correlations between behavior and D2R in both striatal and midbrain regions independent of BMI, our data indicate that D2R levels within this reward pathway may reflect degree of motivation for or sensitivity to obtaining non-food reward and alleviating negative emotions via eating in normal-weight and obese individuals. However, our findings should be interpreted with caution since they are correlational and future studies may experimentally test this hypothesis and alternative explanations.

Our moderately obese participants self-reported higher rates of emotion- and reward-based eating behavior but less non-food reward behavior relative to normal-weight individuals. Obese individuals also tended to self-report sensitivity to punishment to a greater extent than normal-weight individuals. Other studies also show higher rates of eating due to emotional distress in obesity^7^,^47^,^48^,^49^,^50^ as well as positive correlations between food-related reward behavior and BMI^26^,^51^,^52^,^53^. However, our results contrast with a previous study that showed an inverse relationship between BMI and non-food reward behavior in obese people^54^. Although our obese group reported lower rates of non-food reward behavior relative to the normal-weight group, BMI was still positively related to non-food reward behavior within obese participants. One possible explanation for our finding is that while moderately obese individuals self-report reduced non-food reward-based behavior relative to normal-weight participants, there remains a gradient in which both food and non-food reward sensitivity is greater in obese individuals with higher BMI. Alternatively, there may be reward-insensitive and reward-sensitive subtypes of moderate obesity. Finally, few studies have evaluated punishment-related behavior in obesity but Franken and Muris^55^ found no significant correlation between sensitivity to punishment and food craving in participants ranging from underweight to obese while another study demonstrated lower behavioral inhibition in obese individuals^7^. Taken together, our behavioral findings support the idea that obese individuals may experience ‘reward deficiency syndrome’^56^, in which over-consumption of food may compensate for reduced ability to experience pleasure from other activities. Alternatively, RDS in obesity may be secondary to stronger hedonic response to food in individuals with enhanced striatal DA function^37^, putting them at risk of overeating and eventually overriding desire for other rewarding stimuli. Longitudinal investigation of the effect of intervention-induced changes in BMI on reward-related behavior will help clarify this relationship.

There are some limitations to the current study. First, we urge caution in interpreting our findings regarding relationships between central D2R binding and behavior because, admittedly, several hierarchical linear regression analyses were performed without stringent multiple comparisons correction. However, our findings are supported by previous studies: Guo *et al.*^9^ detected a relationship of a similar nature between dorsal D2/D3R binding and ‘opportunistic eating’ and the midbrain is known to function as a modulator of motivation for food and non-food reward^39^,^40^,^57^. Still, due to the novel nature of our findings and the small sample on which they are based, these results will require replication. Further, we did not find any specific clusters of D2R binding within striatum or midbrain that related to eating or reward-based behavior. Our voxelwise analyses was likely less sensitive to these relationships due to variability in D2R binding at the voxelwise level; in contrast, the ROI-based analyses reduced variability in these measures due to use of mean binding potential across regions that were eroded to minimize partial volume effects of neighboring regions known to have less D2R binding. Second, our results cannot explain whether emotional eating or non-food reward behavior precedes higher central D2R binding or vice versa, a key question in terms of understanding, preventing, or treating obesity. Also, due to time constraints, we did not control for whether participants were fasted or sated while completing relevant questionnaires and computer tasks. While this is an important factor to control for in the future, we cannot know how hunger state may have affected our results here since we did not ask participants to rate satiety. In regards to the PET scan, [^11^C]NMB is not displaceable by endogenous DA and therefore D2R binding potential should not be impacted by satiety state. Finally, this study was designed to obtain baseline striatal D2R binding in normal-weight and obese individuals unconfounded by health conditions and medications that interact with or affect DA signaling. Consequently, our results do not generalize to normal-weight or obese individuals with clinical diagnosis-level mental disorders thought to underlie some types of eating behaviors that may involve DA signaling including depression, impulsivity, binge eating and substance abuse. Effects of the interactions between obesity and these disorders on central D2R are highly important and merit further investigation. Despite these limitations, our results offer a template for testable hypotheses that address the limitations described.

In summary, relative to the normal-weight group, the obese group self-reported lower rates of non-food reward behavior and higher rates of eating behavior related to negative affect, sensitivity to rewarding properties of palatable foods, and sensitivity to punishment. Self-reported emotional eating positively correlated with striatal and midrain D2R binding across normal-weight and obese individuals. Higher rates of self-reported non-food reward-related behavior were associated with higher midbrain D2R binding. Taken together, our findings indicate that there are fundamental differences in self-reported eating and reward-related behavior between normal-weight and obese individuals and that, across both groups of individuals, D2R binding levels in the mesostriatal DA system may reflect degree of motivation to alleviate negative emotion through eating and for obtainment of non-food reward. Longitudinal investigations of how these variables interact and contribute to excessive body weight will help identify potential pharmacological and behavioral targets for prevention and/or treatment of obesity.

## Additional Information

**How to cite this article**: Eisenstein, S. A. *et al.* Emotional Eating Phenotype is Associated with Central Dopamine D2 Receptor Binding Independent of Body Mass Index. *Sci. Rep.* **5**, 11283; doi: 10.1038/srep11283 (2015).

## Figures and Tables

**Table 1:** Participant Characteristics

	Normal-weight	Obese
Gender Distribution Ethnicity Distribution	4 men, 13 women 16 C, 1 AA	3 men, 19 women 11 C, 10 AA, 1 Bi*
	Mean (SD), range	
Age (years)	28.5 (5.5), 21–39	31.4 (6.3), 23–40
Education level (years)	16.6 (1.4), 14–19	15.1 (1.9)*, 12–18
Body Mass Index (kg/m^2^)	22.1 (2.0), 18.7–25.9	39.6 (5.2)***, 33.4–51
Beck Depression Inventory II	3.3 (4.4), 0–15	6.0 (6.1), 0–21
IQ	113.0 (12.7), 87–131^a^	107.5 (12.3), 84–131

C, Caucasian; AA, African American; Bi, Biracial

*, *p* < 0.05, ***, *p* < 0.001 relative to normal-weight group

^a^Data not available for 2 participants

**Table 2:** Behavioral Domains. Normal-weight *n* = 17; Obese *n *= 21-22.

Domain and Included Questionnaires	Normal-weight	Obese
**Eating Related to Emotion**	−1.46 (1.86)	1.13 (2.72)**
Emotional Eating Scale total score (EES, Arnow *et al.*, 1995)	12.53 (13.13)	27.59 (18.95)**
Dutch Eating Behavior Questionnaire Emotional Scale average score (DEBQ ES, van Strien *et al.*, 1986)	1.60 (0.57)	2.51 (0.97)**
Sweet Taste Questionnaire Mood Altering Effects (STQ MAE, Kampov-Polevoy *et al.*, 2006) total score	17.82 (7.27)	23.82 (6.97) ^†^
**Eating Related to Reward**	−1.83 (1.83)	1.42 (1.90)***
Binge Eating Scale total score (BES, Gormally *et al.*, 1982)	6.00 (4.37)	15.09 (6.70)***
Sweet Taste Questionnaire Impaired Control over Eating Sweets (STQ IC) total score	14.47 (5.97)	21.68 (5.45)***
Food Craving Inventory total of averaged scores (FCI, White *et al.*, 2002)	7.95 (2.48)	10.28 (2.05)**
**Reward**	0.82 (2.22)	−0.66 (.2.26)*
Behavioral Activation System (BAS) total score from BIS/BAS (Carver and White, 1994)	41.71 (4.73)	37.48 (5.76)*
Reward expectancy total score from Generalized Reward and Punishment Expectancy Scales (GRAPES, Ball and Zuckerman, 1990)	8.71 (2.80)	7.24 (3.38)
Sensitivity to reward total score from Sensitivity to Punishment and Reward Questionnaire (SPSRQ, Torrubia *et al.*, 2001)	8.53 (3.00)	8.62 (3.58)
Summed curious, reward dependency, and persistence scores from Temperament and Character Inventory (TCI-R, Cloninger *et al.*, 1994)	43.47 (7.37)	41.57 (5.27)
**Punishment**	−1.02 (2.46)	0.82 (3.85)^†^
Behavioral Inhibition System (BIS) total score from BIS/BAS	19.41 (3.45)	20.91 (4.33)^†^
Punishment expectancy total score from GRAPES	6.35 (2.55)	7.43 (2.75)
Sensitivity to punishment total score from SPSRQ	6.76 (4.07)	9.45 (6.68)
Harm avoidance total score from TCI-R	10.76 (4.25)	13.68 (6.38)^†^

Summed *z*-scores (S.D.) shown for each domain. Average score (S.D.) shown for individual questionnaires. ***, **, *, *p* ≤ 0.001, 0.01, 0.05 relative to normal-weight group. ^†^, *p* = 0.06 relative to normal-weight group.
